# Three-Dimensional Multimodality Image Reconstruction as Teaching Tool for Case-based learning among medical postgraduates: a focus on primary pelvic bone Tumour Education

**DOI:** 10.1186/s12909-023-04916-8

**Published:** 2023-12-12

**Authors:** Xin Hu, Yitian Wang, Jian Li, Ping Qing, Xiao Yang, Jing Zeng, Li Min, Chongqi Tu

**Affiliations:** 1 Department of Orthopedic Surgery and Orthopedic Research Institute, West China Hospital, Sichuan University, Chengdu, Sichuan 610041 China; 2 Educational Department of Internal Medicine, West China School of Medicine, Sichuan University, Chengdu, Sichuan 610041 China; 3 Department of Cardiology, West China Hospital, Sichuan University, Chengdu, Sichuan 610041 China; 4 Department of Medical Education, West China Medical Center, Sichuan University, Chengdu, Sichuan 610041 China; 5 National Engineering Research Center for Biomaterials, Sichuan University, Chengdu, Sichuan 610064 China; 6 Provincial Engineering Research Center for Biomaterials Genome of Sichuan, Sichuan University, Chengdu, Sichuan 610064 China

**Keywords:** Medical College, Postgraduates, Case-based learning, Pelvis, Oncology

## Abstract

**Background:**

Postgraduate medical education in oncology orthopedics confronts obstacles when instructing on pelvic tumors, primarily due to their intricate anatomy and the limitations of conventional teaching techniques. The employment of Three-dimensional multimodality imaging (3DMMI) can be considered a valuable teaching tool, as it gracefully elucidates the intricacies of pelvic anatomical structures and the interactions between tumors and surrounding tissues through three-dimensional imaging, thereby providing a comprehensive and nuanced perspective. This study aimed to assess the feasibility and effectiveness of incorporating 3DMMI in combination with a Case-Based Learning (CBL) approach for postgraduate education.

**Methods:**

The study encompassed a 10-week course involving 90 surgical postgraduates, focusing on common pelvic tumor diseases. Students were assigned representative clinical cases, and each group created a PowerPoint presentation based on these cases. The core educational content included fundamental knowledge of pelvic anatomy, as well as clinical presentations, radiological features, and treatment principles of common pelvic tumor diseases. The research compared two groups: a traditional CBL group (n = 45) and a 3DMMI-CBL group (n = 45). The 3DMMI-CBL group had access to advanced imaging technology for better visualization. Various evaluations, including image interpretation, theoretical knowledge, and questionnaires, were used to assess the learning outcomes.

**Results:**

The 3DMMI-CBL group outperformed the CBL group not only in the imaging diagnosis of common pelvic diseases but also in their mastery of the related theoretical knowledge. Student questionnaires indicated higher scores for the 3DMMI-CBL group in basic pelvic anatomy knowledge (8.08 vs. 6.62, *p* < 0.01), image interpretation (8.15 vs. 6.69, *p* < 0.01), learning efficiency (8.07 vs. 7.00, *p* < 0.01), clinical reasoning (7.57 vs. 6.77, *p* < 0.01), and learning interest (8.46 vs. 7.00, *p* < 0.01). Teacher questionnaires revealed that 3DMMI technology enhanced teachers’ clinical knowledge, facilitated instruction, and increased overall satisfaction and interest in teaching.

**Conclusion:**

Our study introduced an enhancement to the conventional Case-Based Learning (CBL) model by incorporating 3DMMI technology for visualizing pelvic anatomy. In contrast to pure CBL, this adaptation improved teacher instruction, substantially heightened student engagement, ignited greater interest in learning, and boosted overall efficiency, ultimately leading to positive learning outcomes. Consequently, our study demonstrated the potential feasibility and acceptability of the 3DMMI-CBL teaching method for postgraduates in pelvic bone tumor education.

**Supplementary Information:**

The online version contains supplementary material available at 10.1186/s12909-023-04916-8.

## Introduction

Postgraduate medical education serves as a cornerstone in the comprehensive training of physicians [1]. With the continuous development and transformation of medical education, the number of postgraduate medical students has been increasing year by year in China, especially in oncology orthopedics. Orthopedic oncology is a specialized medical field dedicated to the diagnosis, treatment, and prevention of diseases concerning both primary and metastatic tumors affecting the entire skeletal system. In the field of oncology orthopedics, the diagnosis and surgical treatment of pelvic tumors have always been one of the greatest challenges [2]. These difficulties can largely be attributed to the complex anatomy of the pelvis, which encompasses major blood vessels, nerves, and crucial organs like the ureters, bladder, and bowel [3]. The close proximity of these structures within the pelvic cavity further complicates the process. Moreover, pelvic tumors exhibit insidious onset, wherein significant symptoms usually manifest only when the tumor has already progressed to a considerable size, resulting in extensive infiltration of surrounding tissues [4, 5]. This intricate scenario substantially amplifies the complexity associated with both diagnostic procedures and treatment interventions. Pelvic bone tumor, with all its characteristics, also presents itself as a challenging subject to learn. Postgraduates must transition from passive to active learning methodologies and employ more effective teaching methods when dealing with this complex subject.

The undeniable influence of psychological factors such as self-efficacy, motivation, and attitude on accomplishing goals has been extensively revealed by the psychological research [6]. However, the conventional pedagogy, characterized by a teacher-centered, class-oriented, and examination-driven approach, tends to render students passive recipients rather than active participants in the learning process [7]. In contrast, Case-Based Learning (CBL) is an instructional method that positions the student in a central role, a strategy that has demonstrated significant effectiveness, particularly for small groups of medical students in postgraduate education and for professional development purposes [8, 9]. For example, before engaging in the CBL tutorial, students are required to extensively prepare themselves by acquiring a solid understanding of the background knowledge pertaining to the assigned case [10]. This proactive approach significantly enhances students’ motivation when tackling the challenges associated with learning such a complex subject. Although the use of CBL alone in the field of oncology orthopedics may not be highly effective in promoting knowledge acquisition and student engagement [11], this is especially noticeable in courses that concentrate on pelvic bone tumors. The reasons for this may be attributed to the inherent complexity of these subjects, which can impede the learning process for students from the very beginning. Traditional teaching methods rely on the interpretation of two-dimensional X-ray images or pelvic CT and MRI scans to understand the radiological manifestations and anatomical structures of pelvic tumors. Even for young specialists, it can be challenging, and for graduate students, the difficulty of learning is self-evident.Moreover, students may perceive classroom CBL activities as time-consuming [12] and may experience discomfort when participating in group learning settings, often preferring individual work. These factors collectively have the potential to diminish learning outcomes in this context.

Since the rapid development of computer science and image alignment algorithms in the 1990s, clinical medicine has been profoundly influenced by fusion imaging technology, especially three-dimensional multimodality image (3DMMI) [13]. It leverages the benefits of various imaging technologies and objectively presents integrated three-dimensional reconstructed images on the computer, eliminating the need for mental visualization. Currently, 3DMMI has gained significant traction in supporting various clinical practices, such as precise tumor radiotherapy [14], brain tumor neuro-navigation [15], and preoperative planning for bone tumor resection [16]. Given its remarkable characteristics, 3DMMI holds great potential as an educational resource, enabling students to comprehend complex pelvic structures and the imaging features of pelvic tumors with ease. As a result, this approach can effectively reduce students’ pre-CBL apprehension, cultivate a positive learning attitude, and boost their self-efficacy. It might serve as a valuable tool for CBL in the context of pelvic bone tumors, effectively surmounting the physical constraints associated with traditional CBL methods.

This study is designed to examine the feasibility and acceptance of an innovative pedagogical method known as 3DMMI-CBL within the context of pelvic bone tumors. The study primarily targets postgraduate students specializing in surgery, aiming to investigate how this method can enhance students’ attitudes, self-efficacy, and motivation. Real clinical cases will be used as demonstrative examples to showcase the effectiveness of 3DMMI-CBL.

## Methods

### Course schedule and Pedagogy Method

**Fig. 1:**
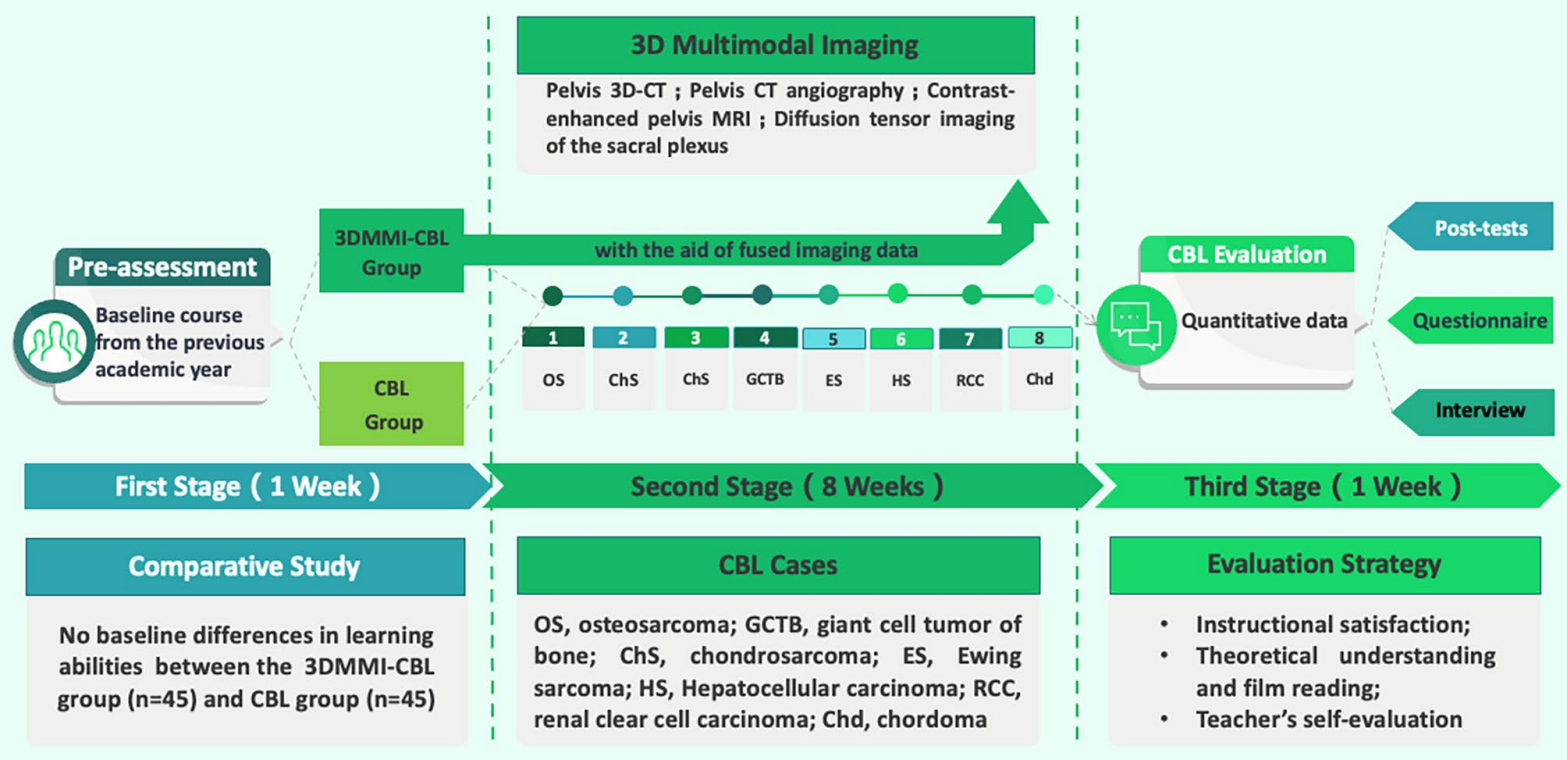
Basic flowchart of the 3DMMI-CBL and CBL teaching modes

The basic flowchart of the comparison between CBL and 3DMMI-CBL mode is shown in Fig. 1. The course extended over a duration of 10 weeks, encompassing a total of 35 instructional hours: 1 week for pre-tests, 8 weeks for implementation, and 1 week for post-tests. A total of 8 comprehensive learning sessions were conducted, encompassing clinically authentic cases of primary and metastatic pelvic tumors. These cases were carefully selected to cover a diverse range of pathological diagnostic types and anatomical locations of pelvic tumors (Table 1). The course was designed with a primary emphasis on five pivotal areas, specifically: (1) pelvic anatomy, (2) characteristics of lesions, (3) interpretation and diagnosis of imaging, (4) selection of appropriate treatment, and (5) fundamental surgical principles.

**Table 1:** Course Content and Basic Information about the Cases

Patient	Age	Gender	BMI	Diagnosis	Enneking staging	Tumor location^a^	Neoadjuvant chemotherapy	Therapy
1	53	F	19	Osteosarcoma	III	PII + III	Two cycles	ERB + ER
2	40	M	24	Chondrosarcoma	IIB	PI + II	No	ERB + ER
3	43	F	20	Chondrosarcoma	IIB	PII	No	ERB + ER
4	34	M	25	Giant cell tumor of bone	/	PII + III	No	ERB + ER
5	18	F	20	Ewing sarcoma	IIB	PI	Two cycles	ERB + ER
6	54	M	22	Hepatocellular carcinoma	/	PII	No	ERB + ER + PLT
7	52	F	23	Renal clear cell carcinoma	/	PII + III	No	ERB + ER + PLT
8	41	F	21	chordoma	IIB	PI + II + III	Two cycles	ERB + ER

*BMI, body mass index;* ^a^ *According to Enneking and Dunham; ERB, En bloc resection for tumor; ER, endoprosthetic reconstruction; PLT, primary lesion treatment*

Prior to each teaching session, the Orthopedics Teaching and Research Group would select 2–3 representative clinical cases for that particular round based on the availability of ward resources and the teaching syllabus. Patient medical history, physical examination findings, and imaging data were collected for these cases. All participating students were informed about these cases by the teaching secretary three days in advance. In order to maximize student self-efficacy and foster a positive learning attitude, each group was required to create their own Microsoft PowerPoint presentation (Microsoft Corporation, Redmond, WA, USA) based on the cases presented by the teacher. This process involves consulting relevant medical textbooks and searching for the latest professional literature. During the CBL sessions, each group had a designated time of 5 min to deliver a concise presentation. As a facilitator and motivator of learning within the group, the tutor summarized and provided explanations for any questions raised. After completing the PowerPoint presentations and the tutor’s summary, bedside teaching was conducted. The teaching faculty guided the students in patient interaction, image interpretation, and discussions related to pelvic tumors. The topics covered included pelvic tumor anatomy, extent of involvement, radiological evaluation, diagnosis, classification, treatment options, surgical principles, neoadjuvant chemotherapy, and targeted therapies.

### Participants

In this study, a cohort of 90 participants was meticulously assembled. These individuals, all in their third year of medical postgraduate studies, specialized in the field of surgery at West China School of Medicine, Sichuan University. In accordance with the pedagogical framework of West China School of Medicine, Sichuan University, these students had not hitherto engaged in clinical internships or surgical rotations, nor had they encountered coursework pertaining to the intricacies of pelvic tumor knowledge. The students were randomly assigned to eight balanced gender-stratified internship blocks. This allocation process was accomplished through a completely random method, leveraging the digital number generation capabilities of Microsoft Excel 2010 (Office 360, Microsoft Ltd., USA). Each block consisted of approximately 11–12 students. The baseline course utilized for comparison was the clinical skills course from the previous academic year, encompassing 250 h of instruction and earning 13 credits. Course grades were analyzed using SPSS 20.0, and variance homogeneity was confirmed (p-value = 0.549) through one-way analysis of variance (ANOVA) to ensure no baseline differences in learning abilities among the eight internship groups (F-value = 1.754, *p* = 0.108) (Table 2). Subsequently, four blocks were designated for the 3DMMI-CBL group as the experimental group, while the remaining four blocks were allocated to the traditional CBL group as the control group.

Clinical doctors (n = 4) with a minimum of three years of experience in traditional CBL teaching were chosen as tutors for this study. All orthopedic educators had similar clinical and teaching backgrounds. During the 8-week course duration, two groups of students (CBL and 3DMMI-CBL) underwent weekly learning sessions focused on the same case. Each group received instruction from the same teacher, and the teaching responsibilities were rotated among four instructors on a weekly basis.

This study was conducted during the 2021–2022 academic year. It was performed in accordance with the 1964 Helsinki Declaration and was authorized by the Ethics Committee of West China Hospital. We obtained written informed consent from all participants in this study, including patients, students, and teachers.

**Table 2:** Previous Courses Performance of 3DMMI-CBL and CBL Group

	CBL	3DMMI-CBL	*p*-value
n	45	45	-
Score	86.03 ± 5.08	85.91 ± 4.09	0.899

### Characteristics of 3DMMI-CBL imaging

According to our previous reports, the steps involved in creating the three-dimensional pelvic model used in the 3DMMI-CBL group teaching mainly include the following [17, 18]:

1. **Acquisition and Import of Image Data**: Based on real cases of pelvic tumor patients, data such as preoperative three-dimensional CT of the pelvis, CT angiography of the abdominal and pelvic arteries, contrast-enhanced MRI of the pelvis, and diffusion tensor imaging of the sacral plexus were collected. These data, in digital imaging and communications in medicine (DICOM) format, were sequentially imported into the interactive modeling software Mimics V21.0 (Materialise Corp. Belgium). The Landmark Registration function was utilized to ensure that different imaging data were reconstructed in the same coordinate system.
2. **Reconstruction of the Three-Dimensional Pelvic Model**: Differentiating the bony structures of the pelvis, vascular nerves, tumors, bladder, and urethra, among other anatomical structures, was accomplished using “Global Threshold Segmentation,” “Region Growing,” and “Edit Mask” functions within Mimics V21.0, based on imaging differences in various tissues. The “Calculate Part” function was used to reconstruct a three-dimensional imaging model, with the model quality set to “Optimal.”
3. **Post-Processing and Visualization**: The obtained three-dimensional pelvic model in Stl format was imported into the three-dimensional model analysis software Geomagic Studio 2014 (3D Systems, Inc. USA). A series of operations in the “Polygons” module, including “Remesh,“ “Remove Spikes,“ “Sandpaper,“ and “Defeature,“ were performed to enhance the quality of the three-dimensional model. The model was saved in Stl format and imported back into Mimics V20.0 software to complete the final reconstruction of the three-dimensional pelvic tumor model. The 3D multimodal imaging (3DMMI) facilitated the integration of all pelvic organs and tissues, including primary pelvic bone sarcomas, adjacent organs, blood vessels, and nerves. Additionally, the transparency of various segments of the three-dimensional model can be adjusted, cross-sectional operations can also be performed, and the intricacies of the three-dimensional reconstruction model can be examined from various perspectives to facilitate optimal observation and learning.

**Fig. 2:**
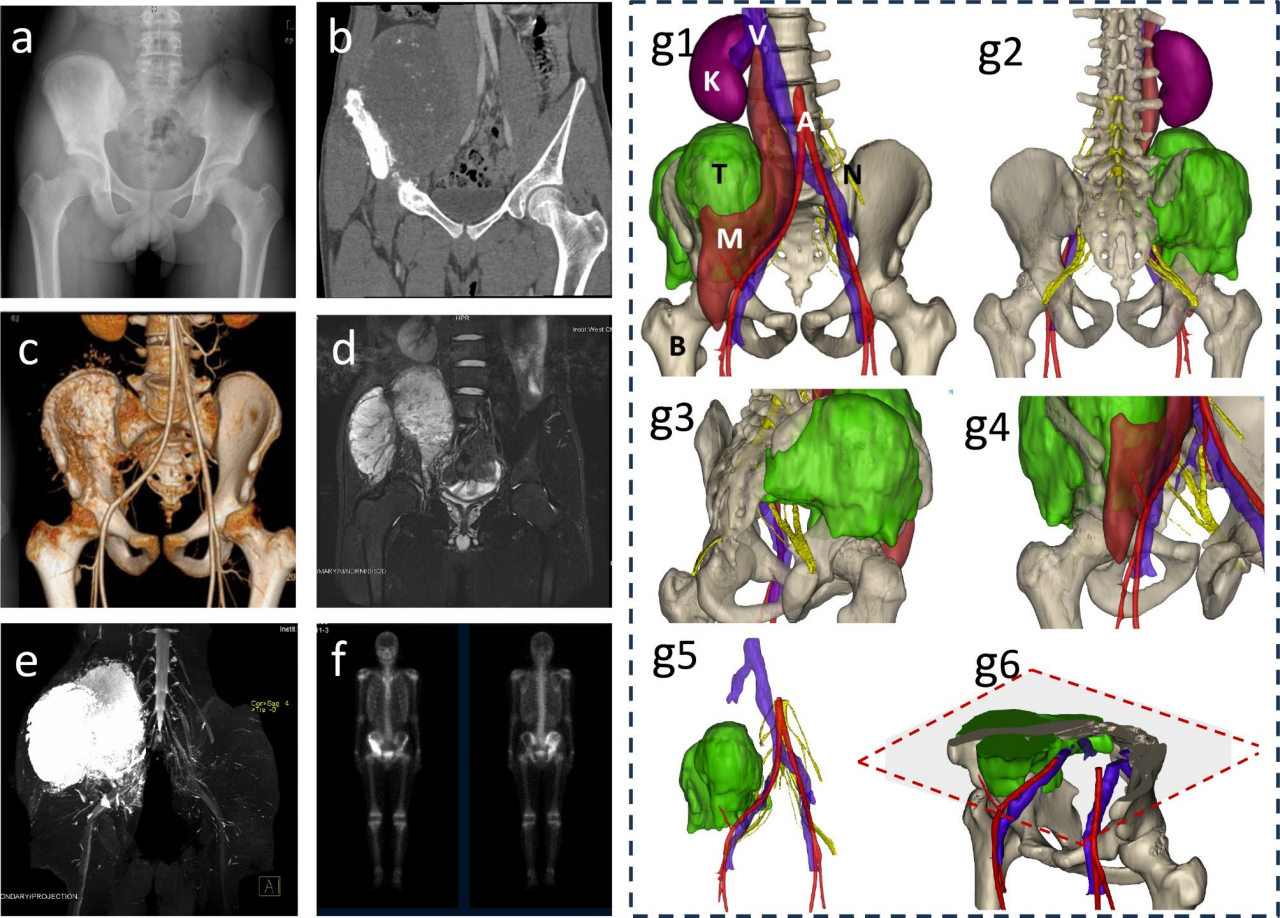
**The 3DMMI fusion image of a typical pelvic bone tumor case: (a)** preoperative pelvic X-ray image, **(b)** Pelvic 3D CT, **(c)** 3D-CT angiography, **(d)** contrast-enhanced MRI displaying the tumor and its soft tissue boundaries, **(e)** diffusion tensor imaging of the sacral plexus, and **(f)** whole-body nuclear medicine bone scanning (ECT). **(g1)** The anterolateral view displays a lesion extending from the periacetabulum to the level of the kidney, involving the sacrum. The right external iliac vessels and ureter remain unaffected due to the protection provided by the iliacus muscle and psoas major muscle. Abbreviations: K, kidney; M, muscle; N, nerve; T, tumor; V, vein; A, artery; B, bone. **(g2-3)** The posterior perspective images using pelvic transparency techniques, allowing clear visualization of internal anatomical structures within the pelvis. A significant tumor is observed, with penetration of the second and third sacral nerves, indicating neural encroachment. **(g4)** The 3D reconstructed image displays the relationship between the anterior thigh blood vessels and the tumor. **(g5)** Hide other non-interest areas and observe the relationship between the tumor and blood vessels and nerves. **(g6)** Observe the relationship between the tumor and other surrounding structures through cross-sectional views

The 3DMMI materials will be provided to all students in the 3DMMI-CBL group during the teaching process, accessible through digital devices such as laptops or smartphones (Fig. 2). An additional movie file shows this in more detail [see Additional file 1]. In contrast, the CBL group will only receive conventional radiological data from the same cases.

**Fig. 3:**
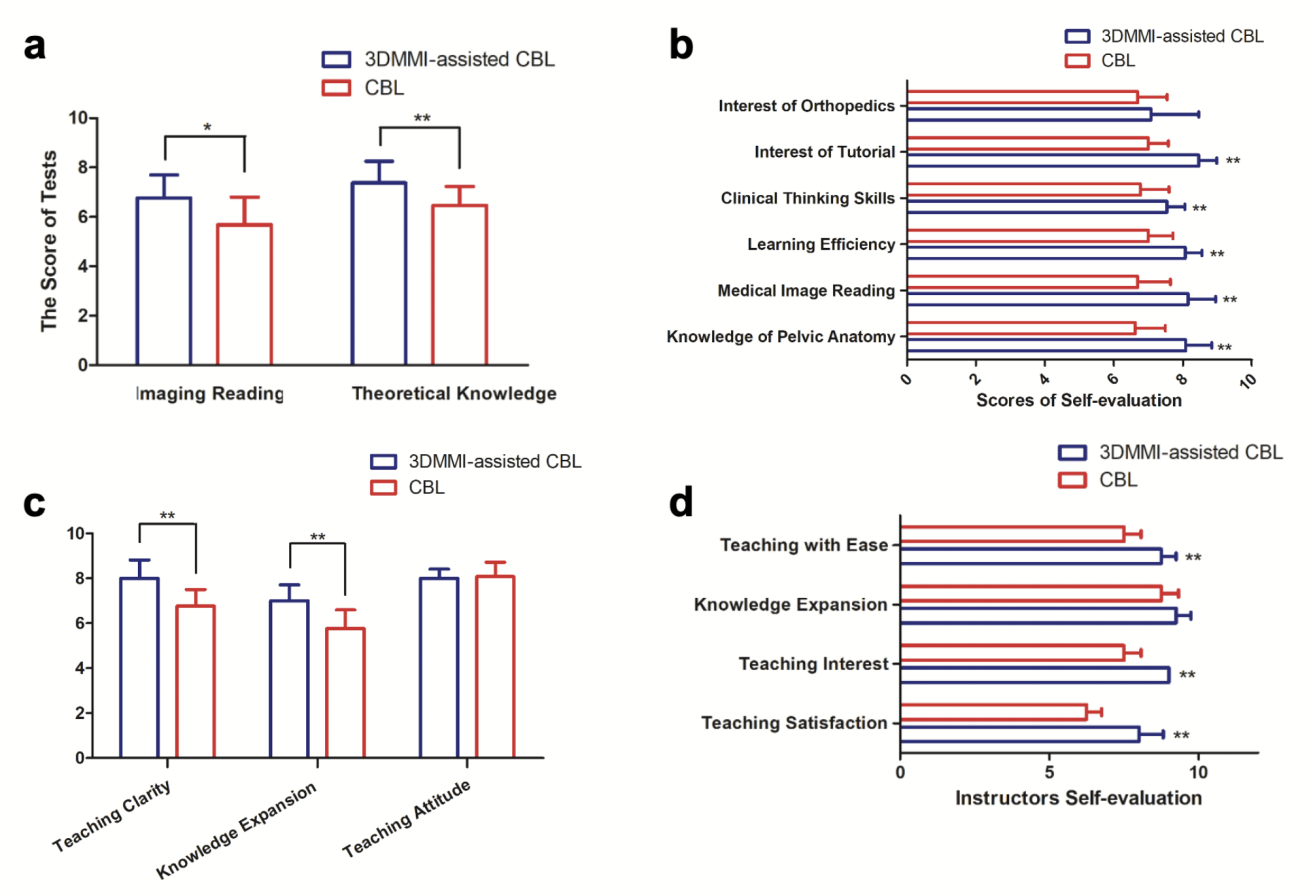
Evaluation and comparison of teaching quality: **(a)** The assessment of knowledge acquisition revealed that the 3DMMI-assisted CBL group attained notably higher scores in both imaging interpretation and theoretical knowledge (*p* < 0.05*, *p* < 0.01**). **(b-c)** The findings from anonymous questionnaires completed by postgraduates indicated that those in the 3DMMI-assisted CBL group exhibited higher scores in several aspects, except for the interest in orthopedics and teaching attitudes of faculty instructors (*p* < 0.05*, *p* < 0.01**). **(d)** The results of instructor self-evaluation demonstrated that teaching with 3DMMI techniques was perceived as less challenging by the instructors. Additionally, they reported higher levels of satisfaction and interest in the 3DMMI-assisted tutorial (*p* < 0.05*, *p* < 0.01**)

### Evaluation system for Course Learning outcomes

This mixed-methods study employs a quantitative approach to evaluate the effects of educational materials developed using 3DMMI technology on student motivation, attitude, and self-efficacy in an orthopedic oncology course [19–21]. Quantitative data is obtained through the following strategies: (1) Evaluation of students’ learning outcomes: This encompasses the assessment of their capacity to interpret imaging data for prevalent pelvic tumor diseases, which involves pelvic X-rays, three-dimensional CT scans of the pelvis, and pelvic MRI, in addition to evaluating their theoretical knowledge. Each component is scored on a scale of 1 to 10. (2) Anonymous questionnaire survey: Conducted among students, it covers course evaluation and teacher evaluation. The course evaluation assesses understanding of pelvic tumors, ability to interpret imaging studies, learning efficiency, clinical thinking abilities, and their interest in the field of orthopedic oncology. The teacher evaluation examines teaching attitude, clarity of knowledge delivery, and knowledge extension. Each component is scored on a scale of 1 to 10 (Additional file 2). (3) Teacher evaluation after each teaching session: Completed by four teachers, it assesses ease of explanation, teaching satisfaction, level of assistance in understanding clinical theory, and teaching interest. Each item is scored on a scale of 1 to 10 (Additional file 3).

### Statistical analysis

The baseline of each postgraduate was compared through one-way ANOVA. The Shapiro-Wilk test verified the normality of continuous data. Normally and abnormally distributed parameters were assessed by the paired two-tailed t-test and Kruskal-Wallis H test, respectively. A p-value of < 0.05 was determined to be statistically significant. Comparisons were conducted between the 3DMMI-CBL group and CBL group by the paired student’s test. The results of questionnaires were analyzed by the Wilcoxon rank-sum test. Data analyses were performed using SPSS 20.0 software (IBM Corporation, Armonk, NY, USA).

## Results

### CBL cases

In our study, a comprehensive set of eight authentic clinical cases was presented to both the CBL group and the 3DMMI-CBL group. The diagnostic spectrum of these cases encompassed a range of pathologies, including giant cell tumor, osteosarcoma, osteochondroma, chordoma, Ewing sarcoma, hepatocellular carcinoma, and renal clear cell carcinoma. Notably, the cases specifically explored tumor localization within the pelvis, employing the Enneking and Dunham classification system [22] to identify various regions, such as Zone I, Zones I + IV, Zone II, Zones I + II, Zones II + III, and Zones I + II + III. These findings are summarized in Table 1.

### Evaluation of CBL and 3DMMI-CBL

According to the post-assessment results of the students, it was found that the 3DMM-CBL group outperformed the CBL group in both clinical skills related to the interpretation of pelvic tumor images and theoretical knowledge assessment of pelvic tumor diseases. The differences in scores were statistically significant, with p-values less than 0.05 (Fig. 3a).

Based on the results of student questionnaires, a total of 90 questionnaires were collected, resulting in a 100% response rate as the questionnaires were distributed and collected simultaneously with the in-class tests. Regarding the course itself, when considering Fig. 3b, it is evident that the 3DMM CBL group achieved significantly higher scores compared to the CBL group in terms of their proficiency in pelvic anatomy knowledge (8.08 vs. 6.62, *p* < 0.01), proficiency in radiographic image interpretation (8.15 vs. 6.69, *p* < 0.01), learning efficiency (8.07 vs. 7.00, *p* < 0.01), clinical reasoning ability (7.57 vs. 6.77, *p* < 0.01), and interest in classroom learning (8.46 vs. 7.00, *p* < 0.01). These differences were statistically significant. However, in terms of interest in orthopedic oncology, although the 3DMM CBL students provided higher ratings, the difference was not statistically significant.

Regarding the evaluation of the teaching faculty, both groups of students highly recognized the teaching attitudes of the instructors. The CBL group scored slightly higher, although the difference was not statistically significant (8.08 vs. 8.00, *p* < 0.718). However, the 3DMM CBL students gave significantly higher ratings to the instructors in terms of knowledge expansion (7.00 vs. 5.77, *p* < 0.01) and clarity of instruction (8.00 vs. 6.77, *p* < 0.01) (Fig. 3c).

Regarding the results of the teacher questionnaires, four teachers conducted two sessions of CBL and two sessions of 3DMMBL teaching each. After each session, the teachers completed questionnaires, resulting in a total of 16 teacher questionnaires collected from the four individuals. However, the results of paired t-tests revealed that 3DMM can assist teachers in enhancing their own clinical theoretical knowledge through teaching, making the instruction process easier (8.75 vs. 7.50, *p* = 0.046). As a result, it increases teachers’ satisfaction (8.0 vs. 6.5, *p* = 0.036) and interest in teaching (7.5 vs. 6.25, *p* = 0.032), with statistically significant differences observed in all aspects. Additionally, there was no significant difference in the expansion of lecture content (9.25 vs. 8.75, *p* = 0.186) between the two groups (Fig. 3d).

## Discussion

CBL is a recognized approach that fosters student-centered learning and nurtures self-directed skills. In medical education, CBL has significant implications for enhancing students’ collaborative and communicative abilities. It equips them with interprofessional teamwork experience and effective patient communication. Furthermore, exposure to real clinical cases enhances practical knowledge and creative problem-solving, deepening their understanding of medical complexities [23–25]. With the evolution of IT (Information technology), medical education benefits from a diverse range of tools. Combining CBL with innovative methods produces positive teaching results. For example, Ping et al. [26] investigated a new pedagogical approach named WeChat-PBL that centered around authentic micromedical cases for hematology postgraduate students. As the most popular communication app in China, WeChat enhances the convenience and accessibility of this unique PBL approach, which also offers opportunities to share current information and educational resources. WeChat-PBL employs participatory, interpretive, and interactive teaching strategies that prioritize effectiveness and engagement. Similarly, Ciloglu and colleagues [27] explored the combination of mobile augmented reality (AR) applications with the CBL teaching model and established that this integrative approach presents a promising method for augmenting students’ motivation, self-efficacy, and attitudes in biology education. The results have implications for the development of novel strategies to optimize students’ learning outcomes. However, to the best of our knowledge, our study represents the first integration of 3DMMI technology into the CBL teaching model, expanding the application scope of CBL in orthopedic oncology education. Our findings revealed that postgraduates in the 3DMMI-CBL group outperformed their peers in the traditional CBL group in terms of clinical image interpretation skills, theoretical knowledge of pelvic tumors, learning interest, learning efficiency, and self-evaluation of teaching effectiveness. These outcomes underscore the recognition and effectiveness of the 3DMMI-CBL teaching approach in postgraduate education for pelvic tumors.

The 3DMMI technology combines the benefits of various imaging examinations to visualize intricate pelvic anatomical structures, including the relationship between tumors and critical blood vessels, nerves, and internal organs [28–31]. This visualization, coupled with clear explanations from the teacher, reduces the barrier to understanding pelvic tumor courses and promotes active student engagement in the learning process. This could potentially account for the observed superior levels of student engagement, learning efficacy, learning satisfaction, and teaching satisfaction exhibited by the 3DMMI-CBL group as compared to the conventional CBL instructional approach. Interestingly, in this study, the increased interaction between mentors, students, and patients during bedside teaching yielded unexpected findings. Patients exposed to both 3DMMI and CBL instruction demonstrated a greater inclination to understand their medical condition and surgical options after 3DMMI instruction. Five out of eight patients expressed this perspective to their attending physicians. Given the inherent difficulty for patients without a medical background to comprehend pelvic tumor surgeries, this finding could further substantiate the efficacy of our teaching approach. However, it should be noted that patient comprehension was not the primary focus, and variables such as educational backgrounds and medical knowledge among patients introduce complexities. Nevertheless, this finding serves as anecdotal evidence for the enhanced intuitiveness and instructional efficacy of the 3DMMI approach.

In addition, the assessment of competence plays a vital role in enabling students to identify and respond to their specific learning needs, while providing valuable insights into their actual performance. Examinations not only shape the content of what students learn but also influence their learning methods [32]. The evaluation of examination scores directly reflects students’ learning effectiveness. In our study, following the completion of the course, we conducted clinical image interpretation assessments of authentic cases, including commonly used clinical examinations such as pelvic X-rays, 3D pelvic CT scans, and pelvic MRI scans. Additionally, a theoretical knowledge examination on pelvic tumors was administered. Interestingly, we observed a significant correlation between the examination scores of students who exhibited higher levels of active participation compared to their peers. Specifically, the 3DMMI-CBL group outperformed the CBL group in both assessments, suggesting that students in the 3DMMI-CBL group demonstrated greater learning engagement and self-efficacy. This can be ascribed to the learner-centered instructional pedagogy embedded in the 3DMMI-CBL approach, wherein students assume a central role in their own learning process. Consequently, the integration of 3DMMI fosters the development of essential competencies such as knowledge acquisition, clinical skills, and professional attitudes, which are instrumental in cultivating active, cooperative, and self-directed learners. By bolstering students’ proficiency in these areas, the 3DMMI approach efficaciously enhances learning outcomes, thereby yielding heightened learning effectiveness and achievement.

There were still some limitations in our preliminary research. Firstly, the sample of participants (90 postgraduates and four teachers) was relatively limited in one institution. Further multi-center studies with larger sizes and various kinds of teaching situations should therefore facilitate determining the efficacy of 3DMMI. Secondly, the strength of 3DMMI lies in its capacity to deliver three-dimensional anatomical imaging and simulate virtual surgeries through software. This versatility extends beyond orthopedic oncology to enrich postgraduate education in surgical disciplines grappling with similarly intricate anatomical knowledge, such as spine surgery, neurosurgery, cardiac surgery, urology, and others. In the foreseeable future, the adoption of 3DMMI-CBL could expand into a wider array of academic fields, affording opportunities to validate its efficacy and adaptability in multidisciplinary settings. Thirdly, even though 3DMMI offers students three-dimensional images of pelvic anatomy, virtual surgeries are presently limited to computer or mobile screen interaction. Down the line, the incorporation of 3D printing technology [33], complemented by data reconstructed from 3DMMI, may pave the way for the creation of physical models that students can interact with and learn from directly (Additional file 3). The fourth point underscores that this study encompassed a range of common pelvic diseases in accordance with the curriculum, acknowledging the inherent variations in the level of difficulty associated with learning different diseases. This inherent diversity somewhat increased the heterogeneity of our research. In subsequent studies, we might conduct similar research with advanced graduate students specializing in bone tumors to further substantiate the impact and importance of 3DMMI technology in pelvic tumor education.

## Conclusion

Leveraging the cutting-edge 3DMMI technology, we have undertaken a visual transformation of pelvic anatomy, elucidating the intricate relationships between tumors and adjacent organ tissues within a three-dimensional model. We have seamlessly integrated this advancement into our teaching methodology, providing a combination of CBL with 3DMMI educational approaches for medical education of oncology orthopedics. In contrast to traditional CBL instruction, the 3DMMI-CBL group exhibited notable benefits in terms of student engagement, overall contentment, efficiency, examination outcomes, and teacher contentment. Our results suggest the viability and acceptability of the 3DMMI-CBL teaching approach for postgraduates in pelvic bone tumor education. This approach holds promise as an effective instructional method for surgical graduate education in this area. Nevertheless, additional research may be required to validate and further promote the adoption of this teaching mode for postgraduates.

### Electronic supplementary material

Below is the link to the electronic supplementary material.

Supplementary Material 1: **Additional file-1** Animated presentation of the three-dimensional reconstruction of pelvic tumor anatomical structures: It incorporates preoperative pelvic X-ray images, Pelvic 3D CT, 3D-CT angiography, contrast-enhanced MRI displaying the tumor and its soft tissue boundaries, diffusion tensor imaging of the sacral plexus, and other three-dimensional pelvic anatomical structure reconstructions that affect the examination. It allows the observation of complex anatomical structures and the relationship between the tumor and surrounding tissues from any angle

Supplementary Material 2: **Additional file-2** Student Questionnaire

Supplementary Material 3: **Additional file-3** Teacher Questionnaire

## Data Availability

The datasets used and/or analysed during the current study available from the corresponding author on reasonable request. We have obtained consent from all patients to publish the data related to the patients involved in this study.
