# Supplementary figures and images for: Three-Dimensional Multimodality Image Reconstruction as Teaching Tool for Case-based learning among medical postgraduates: a focus on primary pelvic bone Tumour Education
Source: BMC Med Educ. 2023 Dec 12;23:944. doi: 10.1186/s12909-023-04916-8 (PMC10717389; doi:10.1186/s12909-023-04916-8)

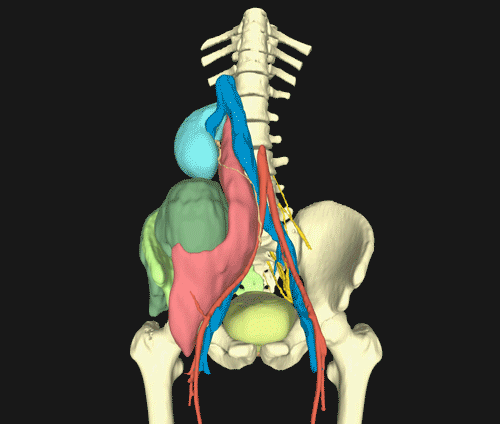

Supplement: Supplementary file 1 — Supplementary Material 1: Additional file-1 Animated presentation of the three-dimensional reconstruction of pelvic tumor anatomical structures: It incorporates preoperative pelvic X-ray images, Pelvic 3D CT, 3D-CT angiography, contrast-enhanced MRI displaying the tumor and its soft tissue boundaries, diffusion tensor imaging of the sacral plexus, and other three-dimensional pelvic anatomical structure reconstructions that affect the examination. It allows the observation of complex anatomical structures and the relationship between the tumor and surrounding tissues from any angle [file 12909_2023_4916_MOESM1_ESM.gif]
