# Supplementary material for: Three-Dimensional Multimodality Image Reconstruction as Teaching Tool for Case-based learning among medical postgraduates: a focus on primary pelvic bone Tumour Education
Source: BMC Med Educ. 2023 Dec 12;23:944. doi: 10.1186/s12909-023-04916-8 (PMC10717389; doi:10.1186/s12909-023-04916-8)
Supplement: Supplementary file 2 — Supplementary Material 2: Additional file-2 Student Questionnaire [file 12909_2023_4916_MOESM2_ESM.docx]

**Questionnaire for Clinical Training in Three-Dimensional Multi-Modal Image Reconstruction Technology in Orthopedics at West China School of Clinical Medicine, Sichuan University (For Students)**

**Anonymous Survey:** Conducted among students, the course evaluation assesses the effectiveness of learning common pelvic tumor diseases as specified in the postgraduate surgical teaching outline of West China School of Clinical Medicine, Sichuan University. This includes the mastery of basic knowledge of normal pelvic anatomy, interpretation of imaging data, learning efficiency, clinical thinking ability, classroom learning interest, and interest in the field of orthopedic oncology.

**Survey Date:** 2020 Academic Year

**Survey Participants:** Eight-year postgraduate students at West China School of Clinical Medicine, Sichuan University (third year, first semester)

Thank you for participating in this survey. Your feedback is very important to us and will help us improve our teaching methods. Please take some time to answer the following questions. We guarantee that this survey is completely anonymous and strictly confidential. It will not be disclosed to anyone unrelated to this study.

**Section 1: Personal Information**

Age:

Gender: Male / Female / Other

Academic Level: Assessment score for the previous academic year's "Basic Diagnosis and Treatment of Diseases." Please provide the score certificate provided by the Postgraduate Department of West China School of Clinical Medicine, Sichuan University.

Knowledge of Pelvic Tumor Diseases and Pelvic Anatomy:

**Section 2: Course Evaluation**

2.1 How well do you understand pelvic anatomy? (1 represents very poor, 10 represents excellent)

[Scale] 1 2 3 4 5 6 7 8 9 10

2.2 How well do you understand the course "Teaching of Three-Dimensional Multi-Modal Image Reconstruction Technology in Orthopedic Clinical Training"? (1 represents very poor, 10 represents excellent)

[Scale] 1 2 3 4 5 6 7 8 9 10

2.3 Please evaluate the quality of teaching materials for this course (textbooks, lecture notes, slides, etc.). (1 represents very poor, 10 represents excellent)

[Scale] 1 2 3 4 5 6 7 8 9 10

2.4 How well do you think this course prepares you for clinical training in orthopedics? (1 represents not well, 10 represents very well)

[Scale] 1 2 3 4 5 6 7 8 9 10

2.5 Please evaluate the extent to which this course has helped improve your ability to interpret images. (1 indicates no help at all, 10 indicates extremely helpful)

[Scale] 1 2 3 4 5 6 7 8 9 10

**Section 3: Teacher Evaluation**

3.1 How satisfied are you with your teachers in terms of course content delivery? (1 represents very unsatisfied, 10 represents very satisfied)

[Scale] 1 2 3 4 5 6 7 8 9 10

3.2 Do you think your teachers have sparked your interest in clinical training in orthopedics? (1 represents not at all, 10 represents completely)

[Scale] 1 2 3 4 5 6 7 8 9 10

**Section 4: Learning Efficiency**

4.1 How efficient do you think your learning is in this course? (1 represents very low, 10 represents very high)

[Scale] 1 2 3 4 5 6 7 8 9 10

4.2 Do you have any suggestions or opinions to improve learning efficiency?

[Open-ended question]

**Section 5: Clinical Thinking Ability**

5.1 Do you think this course has improved your clinical thinking ability? (1 represents not at all, 10 represents significant improvement)

[Scale] 1 2 3 4 5 6 7 8 9 10

**Section 6: Interest in Classroom Learning and Orthopedic Oncology**

6.1 Please rate your level of interest in classroom learning in this course. (1 represents very low, 10 represents very high)

[Scale] 1 2 3 4 5 6 7 8 9 10

6.2 Please rate your level of interest in the field of orthopedic oncology. (1 represents very low, 10 represents very high)

[Scale] 1 2 3 4 5 6 7 8 9 10

6.3 Please rate your willingness to consider a career as an orthopedic oncologist. (1 represents very low, 10 represents very high)

[Scale] 1 2 3 4 5 6 7 8 9 10

6.4 Share your future interests or career aspirations in this field.

[Open-ended question]

Thank you very much for your participation. Your feedback will help us continually improve our teaching methods and teaching quality. If you are willing, you can leave your contact information below for further feedback or to receive the latest information. If you have any questions about this survey, please feel free to contact our staff (contact information below).

Email:

Phone Number:

**Contact Staff:**

**Dr. Hu xin:** Orthopedic Oncologist at West China Hospital, Sichuan University; Phone: 13258260603; Email: greathuxin@163.com; Address: West China Hospital, Wuhou District, Chengdu, Sichuan, China

**Dr. Yitian Wang:** Orthopedic Oncologist at West China Hospital, Sichuan University; Phone: 13980095430; Email: wangytbone199@163.com; Address: West China Hospital, Wuhou District, Chengdu, Sichuan, China
