# Supplementary material for: Three-Dimensional Multimodality Image Reconstruction as Teaching Tool for Case-based learning among medical postgraduates: a focus on primary pelvic bone Tumour Education
Source: BMC Med Educ. 2023 Dec 12;23:944. doi: 10.1186/s12909-023-04916-8 (PMC10717389; doi:10.1186/s12909-023-04916-8)
Supplement: Supplementary file 3 — Supplementary Material 3: Additional file-3 Teacher Questionnaire [file 12909_2023_4916_MOESM3_ESM.docx]

**Questionnaire for Clinical Training in Three-Dimensional Multi-Modal Image Reconstruction Technology in Orthopedics at West China School of Clinical Medicine, Sichuan University (For Teachers)**

**Anonymous Survey:** Completed by teachers, this questionnaire assesses the teaching performance of teachers. The course evaluation includes an assessment of the teaching of common pelvic tumor diseases specified in the teaching outline for surgical graduate students at West China School of Medicine, Sichuan University. This assessment covers the ease of explanation, teaching satisfaction, the level of assistance in understanding clinical theory, and teaching interest. Each item is scored on a scale from 1 to 10.

**Survey Date:** Academic Year 2020

**Survey Participants:** Instructors of the course "Three-Dimensional Multimodal Imaging Reconstruction Technology in Orthopedic Clinical Internship" at West China School of Medicine, Sichuan University

Thank you for participating in this survey. Your feedback is highly valuable to us and will help us improve our teaching methods. Please take a moment to answer the following questions. We guarantee that this survey is completely anonymous and confidential, and your responses will not be disclosed to anyone unrelated to this research.

**Section I: Personal Information**

Age:

Gender: Male / Female / Other

**Section II: Course Evaluation**

**2. Ease of Explanation**

2.1 Please evaluate how easy it is for you to understand the course content during the teaching process. (1 indicates very difficult, 10 indicates very easy)

[Scale] 1 2 3 4 5 6 7 8 9 10

Effectiveness of Teaching Aids

2.2 How effective is the use of teaching aids (e.g., visual materials, presentations) in helping you understand the course content? (1 indicates not effective, 10 indicates very effective)

[Scale] 1 2 3 4 5 6 7 8 9 10

**3. Teaching Satisfaction**

3.1 How satisfied are you with your teaching style and methods? (1 indicates very dissatisfied, 10 indicates very satisfied)

[Scale] 1 2 3 4 5 6 7 8 9 10

3.2 Are the teaching materials (e.g., handouts, presentations) well-organized and helpful for your learning process? (1 indicates disorganized and unhelpful, 10 indicates well-organized and helpful)

[Scale] 1 2 3 4 5 6 7 8 9 10

**4. Ability to Understand Clinical Theory**

4.1 Please evaluate your ability to understand clinical theory and its application in clinical cases. (1 indicates not helpful, 10 indicates very helpful)

[Scale] 1 2 3 4 5 6 7 8 9 10

4.2 When seeking clarification on complex clinical cases, do you encourage questions and provide helpful answers? (1 indicates no encouragement and no assistance, 10 indicates strong encouragement and assistance)

[Scale] 1 2 3 4 5 6 7 8 9 10

**5. Interest in the Subject**

5.1 Please evaluate your interest in the subject matter. (1 indicates not interested, 10 indicates very interested)

[Scale] 1 2 3 4 5 6 7 8 9 10

5.2 Does your enthusiasm for the subject matter positively affect your engagement and interest in the course? (1 indicates no effect, 10 indicates a highly positive impact)

[Scale] 1 2 3 4 5 6 7 8 9 10

**6.Additional Comments**

Do you have any additional comments or suggestions regarding the instructor's performance in this course? Please share your feedback and any specific areas that may need improvement.

[Open-ended question]

Thank you very much for your participation. Your feedback will help us continually improve our teaching methods and the quality of education. If you are willing, you can also provide your contact information below for further feedback or to receive updates. If you have any questions about this survey, please feel free to contact the staff (contact information is provided below).

Email:

Phone Number:

**Contact Information:**

Dr. Huxin Hu, Bone Oncologist, West China Hospital of Sichuan University; Phone: 13258260603; Email: greathuxin@163.com; Address: West China Hospital, Wuhou District, Chengdu, Sichuan, China

Dr. Yitian Wang, Bone Oncologist, West China Hospital of Sichuan University; Phone: 13980095430; Email: wangytbone199@163.com; Address: West China Hospital, Wuhou District, Chengdu, Sichuan, China
